# Benzothiazole-Containing Analogues of Triclocarban with Potent Antibacterial Activity

**DOI:** 10.3390/antibiotics10070803

**Published:** 2021-07-01

**Authors:** Alessia Catalano, Antonio Rosato, Lara Salvagno, Domenico Iacopetta, Jessica Ceramella, Giuseppe Fracchiolla, Maria Stefania Sinicropi, Carlo Franchini

**Affiliations:** 1 Department of Pharmacy-Drug Sciences, University of Bari “Aldo Moro”, 70126 Bari, Italy; antonio.rosato@uniba.it (A.R.); lara.salvagno89@gmail.com (L.S.); giuseppe.fracchiolla@uniba.it (G.F.); carlo.franchini@uniba.it (C.F.); 2 Department of Pharmacy, Health and Nutritional Sciences, University of Calabria, 87036 Arcavacata di Rende, Italy; domenico.iacopetta@unical.it (D.I.); jessicaceramella@gmail.com (J.C.); s.sinicropi@unical.it (M.S.S.)

**Keywords:** antibacterial activity, triclocarban, diarylureas, small molecules, benzothiazole derivatives

## Abstract

Triclocarban (TCC) is a polychlorinated, aromatic, antimicrobial agent commercially used since the 1950s in personal care products for the prevention of spoilage and infections. Humans are frequently exposed to TCC due to its widespread use, leading to its substantial release into the aquatic environment. With the recent ban of TCC from some personal care products, implemented in 2016, many replacement antimicrobial compounds have been studied by researchers. Herein, we report the synthesis and biological activity of a series of diarylureas, analogues of TCC that bear the benzothiazole nucleus as one of the two aryl moieties. Among the studied compounds, **2bF** and **2eC** showed the highest antimicrobial activity against *Staphylococcus aureus*, being also more active than TCC, with MIC values of 8 µg/mL versus 16 µg/mL of TCC. Moreover, compound **2bB** was much more active than TCC against *Enterococcus faecalis*, a Gram-positive bacterium that is, unfortunately, strongly responsible for nosocomial infections. Finally, interesting results were found for compound **2bG** that, even though less active than the others, exerts an interesting bactericidal action.

## 1. Introduction

**Figure 1:**
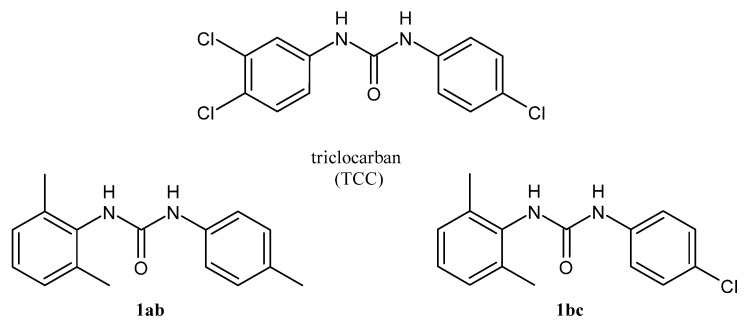
Triclocarban (TCC), and active compounds **1ab** and **1bc**.

Triclocarban (TCC, Figure 1), *N*-(4-chlorophenyl)-*N’*-(3,4-dichlorophenyl) urea, is a polychlorinated aromatic antimicrobial that has been used in personal care products for over 60 years [1] for the prevention of spoilage and infections as it perturbs microbial fatty acid synthesis and cell membrane formation of microbes [2,3]. It belongs to the class of diarylureas (or bis-phenylureas), commonly known for their antitumor properties [4] and numerous other biological activities, and it has been recently proposed for a repositioning to antimicrobial [5] and/or for the treatment of COVID-19 [6]. TCC is added, as a preservative, into consumer products including toothpastes, baby teethers, cosmetics, household sponges, plastic cutting boards, socks and undergarments [7,8]. It is metabolized to hydroxylated species and glucuronides [2,9] and detected in human tissue such as fingernails and body fluids, including blood, urine and seminal plasma [10,11,12]. Nevertheless, given its broad use in personal care products, some concerns about its endocrine disruptive properties were raised [13]. Its halogenated structure is responsible for the lack of biodegradability and its extensive utilization has led to substantial release into the aquatic environment, thus leading to a high environmental risk [14,15]. Recently, it has been suggested that TCC can also affect embryonic development [16]. In September 2016, the U.S. Food and Drug Administration banned its use in over-the-counter wash products, driven by the concern that the use of these products contributes to antibiotic resistance and might negatively affect human health, either through endocrine disruption or modification of the human microbiota [17,18]. Many antimicrobial replacement compounds for TCC have been then used, such as benzalkonium chloride, benzethonium chloride and chloroxylenol. However, it seems that these replacement compounds are not any safer than the banned antimicrobials [19]. Thus, the search for new antimicrobial agents that may substitute TCC and overcome antimicrobial resistance [20,21] is a very important goal to pursue. Recently, we reported a series of small molecules, which are diarylureas, analogues of TCC, and evaluated their antibacterial activities against Gram-positive and Gram-negative bacteria. The most interesting compounds of the series were **1ab** and **1bc** (Figure 1), which showed the same activity of TCC against *Staphylococcus aureus* (MIC = 16 µg/mL) [22]. They both bear the 2,6-xylyl moiety that is found in different compounds with various activities [23,24,25]. The two above-mentioned compounds also showed higher activity than TCC against *Enterococcus faecalis* (MIC = 32 µg/mL versus MIC = 64 µg/mL of TCC), a bacterium notoriously involved in nosocomial infections, which is the third most frequent cause of infective endocarditis (IE), a disease with high morbidity and mortality [26,27].

**Figure 2:**
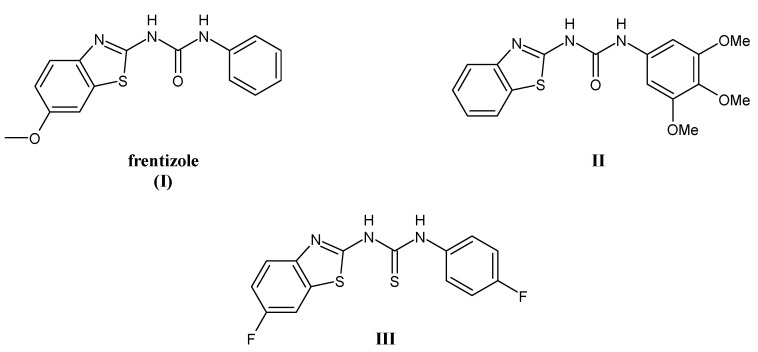
Diarylureas and thioureas.

In the search for new diarylurea analogues more potent as antibacterials than TCC, we sought to analyze new small molecules bearing an aryl moiety different from phenyl. Several nuclei for replacing the phenyl group were considered, finally choosing the benzothiazole (BTA) one that is known to have antimicrobial properties [28,29,30]. BTA is a fused benzoheterocyle present in many naturally occurring and synthetic products and responsible for the medicinal, pharmacological and pharmaceutical applications, including neuroprotective, antidiabetic, antitumor, antimalarial, antitubercular and antimicrobial [31]. Several diarylureas bearing a BTA group have been reported in the literature for diverse biological activities. For instance, frentizole (**I**, Figure 2) acts as an immunosuppressant and is used for the treatment of Alzheimer’s disease (AD), behaving as a weak inhibitor of the Aβ–ABAD interaction (IC_50_ = 200 μM) [32]. Some diarylureas bearing a BTA moiety were studied for the treatment of Parkinson’s disease (PD) as they showed high activity in alleviating haloperidol-induced catalepsy and oxidative stress in mice (**II**, Figure 2) [33]. The thiourea-containing benzothiazole derivative **III,** depicted in Figure 2, showed high antiproliferative activity against the human cell line U-937 (human macrophage cell line) when compared to standard drug etoposide [34].

**Figure 3:**
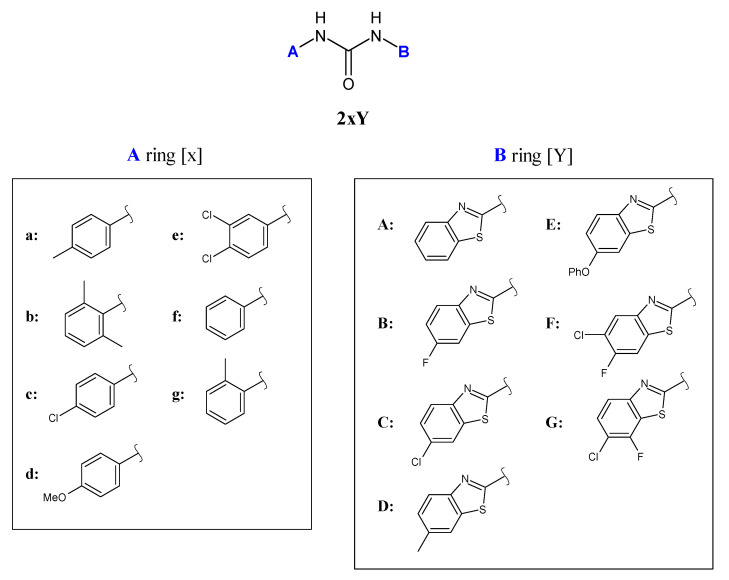
Library composition of diarylureas **2**[x,Y].

Based on the results obtained in our previous paper [22], in this paper, we report the synthesis of several diarylureas (depicted in Figure 3 and detailed in Table 1) by a simple synthetic route, and the study of their antibacterial activity against Gram-positive and Gram-negative strains.

## 2. Results and Discussion

In the search for new antimicrobials more active than TCC, we designed diphenylureas 2xY as benzothiazole analogues of TCC based on our previous results [22]. Several diarylureas, particularly diphenylureas, were tested. Interestingly, compounds 1ab and 1bc (Figure 1) showed the same activity of TCC against *S. aureus* (MIC = 16 µg/mL) and higher activity than TCC against *E. faecalis.*

### 2.1. Chemistry

**Scheme 1:**
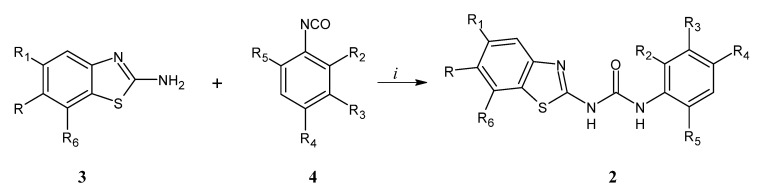
Reagents and conditions: (i) acetone, 100 °C, 6 h.

Diphenylureas **2** were prepared as depicted in Scheme 1. Final products were obtained by reacting the commercial 2-aminobenzothiazoles (**3**) with the appropriate phenylisocyanate (**4**) in acetone, as reported in the literature [35]. Only for compounds **2eE** and **2bF** was the synthesis of the starting material (2-amino-6-phenoxybenzothiazole and 2-amino-5-chloro-6-fluorobenzothiazole, respectively) needed, and it was accomplished as previously reported [36,37].

### 2.2. Antibacterial Studies

A first screening for the antimicrobial activity of our compounds was carried out by means of the Agar diffusion assay [38]. Afterwards, only compounds that showed activity in this test were studied with the microdilution test, using norfloxacin (NRF) as reference. The in vitro minimum inhibitory concentrations (MICs, μg/mL) were assessed by the broth microdilution method according to Clinical Laboratory Standards Institute (CLSI) guidelines [39]. MIC values were recorded as the lowest concentration of compounds at which there was no optically detectable microorganism growth and evaluated by comparing the growth in every well visually with that of the growth control well for bacteria. Compounds were dissolved in dimethyl sulfoxide (DMSO) and tested in a final concentration range from 512 µg/mL to 2 µg/mL. MICs for the reference antibiotic NRF against quality control strains were used to confirm the validity of the screening. According to CLSI guidelines, diphenylureas were tested in vitro against a panel of Gram-positive and Gram-negative bacteria belonging to the ATCC collection, using the standard microdilution test for the determination of MICs as previously reported [40]. Compounds **2eD**, **2dA**, **2aC**, **2gC**, **2fC**, **2bA** and **2bE** demonstrated no activity in the Agar diffusion assay; thus, they were not analyzed via the microdilution assay. MICs of the other compounds, along with the parent compound TCC and the standard antibiotic NRF, are listed in Table 1. Our results showed that the highest activity against *S. aureus* was obtained for compounds **2bF** and **2eC**, showing MIC values of 8 µg/mL, which were lower than the reference TCC (MIC = 16 µg/mL). A slightly lower activity, but on par with TCC, was observed for **2aA, 2eE** and **2bB** (MIC = 16 µg/mL). The other compounds were less active or inactive. Particularly, compound **2bB** interestingly possessed the same activity of TCC against *S. aureus* and showed the highest activity against *E. faecalis* (MIC = 8 µg/mL versus 64 µg/mL of TCC), a commensal and nosocomial pathogen that is difficult to treat [41]. Finally, compound **2bG** was interesting as it exerted bactericidal action. Based on these results, it can be easily deduced that the best substitutions for the aryl moiety are 3,4-dichloro and 2,6-dimethyl. The former is the same as TCC, while the latter confirms our previously reported data [22], corroborating the interesting activity of 2,6-xylyl also in terms of antibacterial activity. Then, the introduction of the BTA as the other aryl moiety leads to compounds with higher activity, especially when it bears halogens, such as chlorine and fluorine, as substituents (see **2bF**, **2eC** and **2bB**). The absence of substituents on the BTA, or the introduction of a bulky group as the phenoxy, does not vary the antimicrobial activity (see **2aA** and **2eE**). Finally, in order to evaluate the cytotoxicity of the most active benzothiazole compounds (**2eC**, **2bG**, **2bF** and **2bB**), we exposed the human non-malignant mammary epithelial MCF-10A cells to increasing concentrations (4, 8, 16, 32 and 100 µg/mL) of the compounds for 24 h. The obtained results showed that **2eC** did not interfere with normal cell growth, at least at the concentrations used in this assay (IC_50_ > 100 µg/mL). Instead, **2bG**, **2bF** and **2bB** exerted a slight cytotoxicity on the non-tumoral cells (IC_50_ = 24.9 ± 1.2, 24.5 ± 0.9 and 27.8 ± 1.0 µg/mL, respectively), even though their IC_50_ values were three-fold higher than their calculated antimicrobial activity (MIC = 8 µg/mL). Thus, at the determined MIC values (shown in Table 1), the most active compounds did not have significant cytotoxic effects against the MCF-10A cells.

## 3. Materials and Methods

### 3.1. Chemistry

All chemicals were purchased from Sigma-Aldrich or Lancaster at the highest quality commercially available. Solvents were reagent-grade unless otherwise indicated. Yields refer to purified products and were not optimized. The structures of the compounds were confirmed by routine spectrometric analyses (^1^H NMR, ^13^C NMR, LC–MS, IR). Only spectra for compounds not previously described are given. Melting points were determined on a Gallenkamp melting point apparatus in open glass capillary tubes and were uncorrected. ^1^H and ^13^C NMR spectra were recorded on a Varian VX Mercury spectrometer operating at 300 and 75 MHz for ^1^H and ^13^C, respectively, or an Agilent 500 MHz operating at 500 and 125 MHz for ^1^H and ^13^C, respectively, using DMSO-*d*_6_ as solvent. Chemical shifts are reported in parts per million (ppm) relative to solvent resonance: *δ* = 2.48 ppm (^1^H NMR) and *δ* = 39.9 ppm (^13^C NMR). *J* values are given in Hz. The following abbreviations are used: s—singlet, d—doublet, t—triplet, dt—double triplet, m—multiplet. Liquid chromatography (LC)–mass spectroscopy (MS) was performed on a spectrometer Agilent 1100 series LC-MSD Trap System VL. The molecular ion was designated as “M^+^”. The infrared spectra were recorded on a Perkin Elmer (Norwalk, CT) Spectrum One FT spectrophotometer and band positions are given in reciprocal centimeters (cm^‒1^).

*1-(3,4-Dichlorophenyl)-3-(6-methyl-1,3-benzothiazol-2-yl)urea (**2eD**)*. A solution of 3,4-dichlorophenyl isocyanate (0.23 g, 1.21 mmol) in acetone (4 mL) was added to a solution of 2-amino-6-methylbenzothiazole (0.20 g, 1.21 mmol) in acetone (4 mL). Resulting mixture was refluxed for 6 h. The solid was filtered to give 0.31 g (73%) of **2eD** as a white solid: mp > 250 °C; LC/MS (70 eV) *m*/*z* (%): 374 [M^+^, +23]; IR (KBr): 3371 (NH), 1623 (C=O) cm^–1^; ^1^H NMR (500 MHz, DMSO-*d*_6_): δ 2.36 (s, 3H, C*H*_3_), 7.18 (dd, *J* = 8.3, 1.0, Hz, 1H, Ar), 7.40–7.51 (m, 2H, Ar), 7.53 (d, *J* = 8.8 Hz, 1H, Ar), 7.65 (s, 1H, Ar), 7.92 (d, *J* = 2.4 Hz, 1H, Ar), 9.49 (s, 1H, N*H*), 11.20 (s, 1H, N*H*); ^13^C NMR (125 MHz, DMSO-*d*_6_): δ 21.3, 117.9, 119.4, 120.3, 121.9, 124.5, 126.9, 127.8, 131.1, 131.5, 132.9, 139.6.

*1-(3,4-Dichlorophenyl)-3-(6-fluoro-1,3-benzothiazol-2-yl)urea (**2eB**).* It was prepared as reported for **2eD** starting from 2-amino-6-fluorobenzothiazole and 3,4-dichlorophenyl isocyanate. Yield: 66% (slightly yellowish solid): mp > 250 °C; LC/MS (70 eV) *m*/*z* (%): 379 [M^+^, +23]; IR (KBr): 3369 (NH), 1712, 1606 (C=O) cm^–1^; ^1^H NMR (500 MHz, DMSO-*d*_6_): δ 7.22 (dt, *J* = 9.2, 2.9 Hz 1H, Ar), 7.43 (dd, *J* = 8.6, 1.9 Hz, 1H, Ar), 7.54 (d, *J* = 8.8 Hz, 1H, Ar), 7.58–7.65 (m, 1H, Ar), 7.80 (dd, *J* = 8.3, 2.4 Hz, 1H, Ar), 7.88 (d, *J* = 1.9 Hz, 1H, Ar), 9.47 (s, 1H, N*H*), 11.22 (br s, 1H, N*H*); ^13^C NMR (125 MHz, DMSO-*d*_6_): δ 108.7, 114.3, 120.0, 124.7, 131.1, 131.6, 139.4, 158.8.

*1-(2,6-Dimethylphenyl)-3-(6-fluoro-1,3-benzothiazol-2-yl)urea (**2bB**).* It was prepared as reported for **2eD** starting from 2-amino-6-fluorobenzothiazole and 2,6-dimethylphenyl isocyanate. Yield: 34% (white solid): mp > 250 °C; LC/MS (70 eV) *m*/*z* (%): 338 [M^+^, +23]; IR (KBr): 3201 (NH), 1682, 1607 (C=O) cm^–1^; ^1^H NMR (300 MHz, DMSO-*d*_6_): δ 2.26 (s, 6H, C*H*_3_), 7.09 (s, 3H, Ar), 7.19 (dt, *J =* 9.1, 2.6 Hz, 1H, Ar), 7.56–7.70 (m, 1H, Ar), 7.78 (dd, *J* = 8.8, 2.6 Hz, 1H, Ar), 8.34 (br s, exch D_2_O, 1H, N*H*), 10.98 (br s, exch D_2_O, 1H, N*H*); ^13^C NMR (125 MHz, DMSO-*d*_6_): δ 108.4, 114.1, 121.2, 127.1, 128.3, 133.2, 134.5, 136.0, 146.3, 152.5, 158.7, 160.3.

*1-(6-Chloro-1,3-benzothiazol-2-yl)-3-(4-chlorophenyl)urea (**2cC**)* [42]. It was prepared as reported for **2eD** starting from 2-amino-6-chlorobenzothiazole and 4-chlorophenyl isocyanate. Yield: 91% (white solid): mp > 250 °C; LC/MS (70 eV) *m*/*z* (%): 336 [M^+^, −1]; IR (KBr): 3234 (NH), 1707, 1598 (C=O) cm^–1^; ^1^H NMR (500 MHz, DMSO-*d*_6_): δ 7.35–7.39 (m, 3H, Ar), 7.52 (d, *J =* 8.8 Hz, 2H, Ar), 7.61 (d, *J* = 8.3 Hz, 1H, Ar*),* 8.02 (s, 1H, Ar), 9.28 (br s, 1H, N*H*), 11.00 (br s, 1H, N*H*); ^13^C NMR (125 MHz, DMSO-*d*_6_): δ 121.0, 121.7, 126.7, 127.2, 127.5, 129.2, 137.7.

*1-(6-Chloro-1,3-benzothiazol-2-yl)-3-(2,6-dimethylphenyl)urea (**2bC**).* It was prepared as reported for **2eD** starting from 2-amino-6-chlorobenzothiazole and 2,6-dimethylphenyl isocyanate. Yield: 62% (white solid): mp > 250 °C; LC/MS (70 eV) *m*/*z* (%): 330 [M^+^, −1]; IR (KBr): 3243 (NH), 1699, 1610 (C=O) cm^–1^; ^1^H NMR (500 MHz, DMSO-*d*_6_): δ 2.19 (s, 6H, C*H*_3_), 7.07 (s, 3H, Ar), 7.37 (d, *J =* 2.4 Hz, 1H, Ar), 7.61 (br d, *J =* 8.3 Hz, 1H, Ar), 8.00 (s, 1H, Ar), 8.34 (br s, exch D_2_O, 1H, N*H*), 11.05 (br s, exch D_2_O, 1H, N*H*); ^13^C NMR (125 MHz, DMSO-*d*_6_): δ 18.6, 121.4, 121.6, 126.6, 127.2, 127.2, 128.3, 133.7, 134.5, 148.4, 152.5, 161.1.

*1-(5-Chloro-6-fluoro-1,3-benzothiazol-2-yl)-3-(2,6-dimethylphenyl)urea (**2bF**).* It was prepared as reported for **2eD** starting from 2-amino-5-chloro-6-fluorobenzothiazole and 2,6-dimethylphenyl isocyanate. Yield: 51% (white solid): mp > 250 °C; LC/MS (70 eV) *m*/*z* (%): 372 [M^+^, +23]; IR (KBr): 3246 (NH), 1609 (C=O) cm^−1^; ^1^H NMR (500 MHz, DMSO-*d*_6_): δ 2.18 (s, 6H, C*H*_3_), 7.11 (s, 3H, Ar), 7.64 (d, *J =* 10.2 Hz, 1H, Ar), 8.13 (d, *J =* 7.3 Hz, 1H, Ar), 8.36 (s, 1H, N*H*), 11.16 (s, 1H, N*H*); ^13^C NMR (125 MHz, DMSO-*d*_6_): δ 18.5, 123.0, 127.2, 128.3, 128.8, 134.4, 136.0, 149.6, 152.4,155.5, 157.4, 163.2.

*1-(1,3-Benzothiazol-2-yl)-3-(4-methoxyphenyl)urea (**2dA**)* [33]. It was prepared as reported for **2eD** starting from 2-amino-benzothiazole and *p*-methoxyphenyl isocyanate. Yield: 59% (white solid): mp > 250 °C, LC/MS (70 eV) *m*/*z* (%): 322 [M^+^, +23]; IR (KBr): 3256 (NH), 1601 (C=O) cm^−1^; ^1^H NMR (500 MHz, DMSO-*d*_6_): δ 3.71 (s, 3H, O-C*H*_3_) 6.89 (d, *J* = 8.8 Hz, 2H, Ar), 7.21 (t, *J* = 7.3 Hz, 1H, Ar), 7.34–7.41 (m, 3H, Ar), 7.62 (d, *J* = 7.8 Hz, 1H, Ar), 7.87 (d, *J* = 7.8 Hz, 1H, Ar), 8.99 (s, 1H, N*H*), 10.78 (br s, 1H, N*H*).

*1-(6-Chloro-1,3-benzothiazol-2-yl)-3-(4-methylphenyl)urea (**2aC**).* It was prepared as reported for **2eD** starting from 2-amino-6-chlorobenzothiazole and *p*-tolylphenyl isocyanate. Yield: 98% (white solid): mp > 250 °C; LC/MS (70 eV) *m*/*z* (%): 316 [M^+^, −1]; IR (KBr): 3215 (NH), 1621 (C=O) cm^−1^; ^1^H NMR (500 MHz, DMSO-*d*_6_): δ 2.25 (s, 3H, C*H*_3_), 7.01 (t, *J* = 7.3 Hz, 1H, Ar), 7.18 (dd, *J* = 7.8 Hz, 2H, Ar), 7.38 (dd, *J* = 8.8, 2.4 Hz, 1H, Ar), 7.62 (d, *J* = 8.8 Hz, 1H, Ar), 7.80 (d, *J* = 7.8 Hz, 1H, Ar), 8.03 (d, *J* = 1.9 Hz, 1H, N*H*), 8.57 (s, 1H, N*H*), 11.2 (br s, 1H, N*H*); ^13^C NMR (125 MHz, DMSO-*d*_6_): δ 18.2, 121.5, 121.6, 121.9, 124.4, 126.6, 126.8, 127.4, 128.8, 130.8, 133.6, 136.5, 148.4, 152.1, 160.7.

*1-(3,4-Dichlorophenyl)-3-(6-phenoxy-1,3-benzothiazol-2-yl)urea (**2eE**).* It was prepared as reported for **2eD** starting from 2-amino-6-phenoxybenzothiazole and 3,4-dichlorophenyl isocyanate. Yield: 46% (white solid): mp > 250 °C; LC/MS (70 eV) *m*/*z* (%): 428 [M^+^, −1]; IR (KBr): 3220 (NH), 1696 (C=O) cm^−1^; ^1^H NMR (300 MHz, DMSO-*d*_6_): δ 6.98 (d, *J =* 8.2 Hz, 1H, Ar), 7.06–7.12 (m, 2H, Ar), 7.32–7.40 (m, 3H, Ar), 7.43 (dd, *J =* 8.8, 2.3 Hz, 1H, Ar), 7.54 (d, *J =* 8.8 Hz, 1H, Ar), 7.62 (t, *J* = 7.6 Hz, 2H. Ar), 7.89 (d, *J* = 2.3 Hz, 1H, Ar), 9.46 (s, 1H, N*H*); ^13^C NMR (125 MHz, DMSO-*d*_6_): δ 112.7, 118.4, 118.8, 119.4, 120.4, 123.5, 124.7, 130.5, 131.1, 131.6, 139.4, 152.6, 158.0.

*1-(6-Chloro-1,3-benzothiazol-2-yl)-3-(3,4-dichlorophenyl)urea (**2eC**)* [42]. It was prepared as reported for **2eD** starting from 2-amino-6-chlorobenzothiazole and 3,4-dichlorophenyl isocyanate. Yield: 75% (white solid): mp ˃ 250 °C; LC/MS (70 eV) *m*/*z* (%): 370 [M^+^, −1]; IR (KBr): 3366 (NH), 1594, 1614 (C=O) cm^–1^; ^1^H NMR (300 MHz, DMSO-*d*_6_): δ 7.37–7.46 (m, 2H, Ar), 7.54–7.61 (m, 2H, Ar), 7.90 (d, *J* = 2.9 Hz, 1H, Ar), 8.03 (d, *J* = 1.8 Hz, 1H, Ar), 9.50 (br s, 1H, N*H*); ^13^C NMR (125 MHz, DMSO-*d*_6_): δ 119.5, 120.5, 121.8, 124.7, 126.7, 127.4, 131.1, 131.6, 139.4.

*1-(6-chloro-1,3-benzothiazol-2-yl)-3-(2-methylphenyl)urea (**2gC**)* [43]. It was prepared as reported for **2eD** starting from 2-amino-6-chlorobenzothiazole and *o*-tolyl isocyanate. Yield: 86% (white solid): mp ˃ 250 °C; LC/MS (70 eV) *m*/*z* (%): 340 [M^+^, +23]; IR (KBr): 3381 (NH), 1594, 1621 (C=O) cm^–1^; ^1^H NMR (300 MHz, DMSO-*d*_6_): δ 2.48 (s, 3H, C*H_3_*), 7.01 (t, *J* = 9.0 Hz, 1H, Ar), 7.37 (dd, *J* = 8.5, 2.0 Hz, 1H, Ar), 7.63 (d, *J* = 8.8 Hz, 1H, Ar), 7.81 (d, *J* = 7.6 Hz, 1H, Ar), 8.03 (s, 1H, Ar), 8.29 (d, *J* = 1.8 Hz, 1H, Ar), 8.56 (br s, exch. D_2_O, 1H, N*H*), 11.2 (br s, exch. D_2_O, 1H, N*H*); ^13^C NMR (125 MHz, DMSO-*d*_6_): δ 18.2, 79.6, 121.6, 121.9, 124.4, 126.6, 126.8, 127.4, 128.8, 130.8, 133.6, 136.5, 148.5, 152.1, 160.7.

*1-(6-Chloro-1,3-benzothiazol-2-yl)-3-phenylurea (**2fC**)* [43]. It was prepared as reported for **2eD** starting from 2-amino-6-chlorobenzothiazole and phenyl isocyanate. Yield: 52% (white solid): mp > 250 °C; LC/MS (70 eV) *m*/*z* (%): 326 [M^+^, +23]; IR (KBr): 3209 (NH), 1566, 1666 (C=O) cm^–1^; ^1^H NMR (300 MHz, DMSO-*d*_6_): δ 7.05 (t, *J* = 7.3 Hz, 1H, Ar), 7.25–7.40 (m, 3H, Ar), 7.49 (d, *J* = 8.2 Hz, 2H, Ar), 7.63 (d, *J* = 8.2 Hz, 1H, Ar), 8.03 (d, *J* = 1.8 Hz, 1H, Ar), 9.14 (s,1H, N*H*), 10.9 (br s, 1H, N*H*); ^13^C NMR (125 MHz, DMSO-*d*_6_): δ 119.3, 121.7, 123.6, 126.7, 127.4, 129.4, 133.4, 138.7, 148.2, 152.2, 160.7, 182.6.

*1-(6-Chloro-1,3-benzothiazol-2-yl)-3-(4-methoxyphenyl)urea (**2dC**)* [43]. It was prepared as reported for **2eD** starting from 2-amino-6-chlorobenzothiazole and 4-methoxyphenyl isocyanate. Yield: 54% (white solid): mp > 250 °C; LC/MS (70 eV) *m*/*z* (%): 356 [M^+^, +23]; IR(KBr): 3307 (NH), 1532, 1682 (C=O) cm^–1^; ^1^H NMR (300 MHz, DMSO-*d*_6_): δ 3.72 (s, 3H, O-C*H_3_*), 6.90 (d, *J* = 8.8 Hz, 2H, Ar), 7.36–7.41 (m, 3H, Ar), 7.61 (d, *J* = 8.8 Hz, 1H, Ar), 8.02 (d, *J* = 2.3 Hz, 1H, Ar), 8.97 (s, 1H, N*H*), 10.81 (s, 1H, N*H*); ^13^C NMR (125 MHz, DMSO-*d*_6_): δ 60.4, 119.3, 126.0, 126.3, 131.4, 132.0, 136.4, 160.5.

*1-(1,3-Benzothiazol-2-yl)-3-(2,6-dimethylphenyl)urea (**2bA**).* It was prepared as reported for **2eD** starting from 2-aminobenzothiazole and 2,6-dimethylphenyl isocyanate. Yield: 19% (beige solid): mp > 250 °C; LC/MS (70 eV) *m*/*z* (%): 320 [M^+^, +23]; IR(KBr): 3385 (NH), 1597, 1698 (C=O) cm^–1^; ^1^H NMR (300 MHz, DMSO-*d*_6_): δ 2.18 (s, 6H, C*H*_3_), 7.10 (s, 3H, Ar), 7.14–7.22 (m, 1H, Ar), 7.25–7.35 (m, 1H, Ar), 7.55–7.70 (m, 1H, Ar), 7.75–7.90 (m, 1H, Ar), 8.43 (br s, 1H, N*H*), 11.0 (br s, 1H, N*H*); ^13^C NMR (75 MHz, DMSO-*d*_6_): δ 18.6 (2C), 121.8, 123.2, 126.3, 127.1, 128.3, 134.6, 136.0.

*1-(2,6-Dimethylphenyl)-3-(6-phenoxy-1,3-benzothiazol-2-yl)urea (**2bE**).* It was prepared as reported for **2eD** starting from 6-phenoxyaminobenzothiazole and 2,6-dimethylphenyl isocyanate. Yield: 50% (beige solid): mp > 250 °C; LC/MS (70 eV) *m*/*z* (%): 388 [M^+^, −1]; IR (KBr): 3266 (NH), 1590, 1635 (C=O) cm^–1^; ^1^H NMR (300 MHz, DMSO-*d*_6_): δ 2.21 (s, 6H, CH_3_), 6.90–7.15 (m, 7H, Ar), 7.36 (t, *J* = 8.0 Hz, 1H, Ar), 7.55–7.70 (m, 1H, Ar); ^13^C NMR (125 MHz, DMSO-*d*_6_): δ 18.6 (2C), 118.3, 123.5, 128.1, 130.5, 136.2, 136.3, 152.4, 158.1.

*1-(6-Chloro-7-fluoro-1,3-benzothiazol-2-yl)-3-(2,6-dimethylphenyl)urea (**2bG**).* It was prepared as reported for **2eD** starting from 6-chloro-7-fluoroaminobenzothiazole and 2,6-dimethylphenyl isocyanate. Yield: 92% (beige solid): mp > 250 °C; LC/MS (70 eV) *m*/*z* (%): 372 [M^+^, +23]; IR (KBr): 3248 (NH), 1593, 1699 (C=O) cm^–1^; ^1^H NMR (300 MHz, DMSO-*d*_6_): δ 2.19 (s, 6H, C*H_3_*), 7.10 (s, 3H, Ar), 7.63 (br d, *J* = 10.5 Hz, 1H, Ar), 8.15 (d, *J* = 7.5 Hz, 1H, Ar), 8.43 (br s, 1H, N*H*); ^13^C NMR (125 MHz, DMSO-*d*_6_): δ 18.6, 123.0, 127.2, 128.3, 134.5, 136.0 152.4, 158.1.

### 3.2. Antibacterial In Vitro Evaluation

The in vitro minimum inhibitory concentrations (MICs, μg/mL) were assessed by the broth microdilution method, using 96-well plates, according to CLSI guidelines (Clinical and Laboratory Standards Institute (CLSI, 2012)) [39]. Stock solutions of the tested compounds were prepared by setting the concentration at the maximum possible value. Then, the stock solutions were diluted 1:10 with Cation-Adjusted Mueller Hinton Broth (Oxoid, Italy). Afterwards, two-fold serial dilutions in the suitable test medium were carried out to obtain a set of concentrations from 512 µg/mL to 2 µg/mL in the wells. To obtain the stock solution, we employed as diluent DMSO 100%. The following bacterial strains, available as freeze-dried discs, belonging to the ATCC collection, were used: Gram-positive strains such as *S. aureus* ATCC 25923, 29213, 6538 and 6538p, *E. faecalis* ATCC 29212, and Gram-negative ones such as *K. pneuomoniae* ATCC 13883, *P. aeruginosa* ATCC 27853. To preserve the purity of cultures and to allow their reproducibility, cryovials of all microbial strains in the medium were set up and stored at −80 °C. Pre-cultures of each bacterial strain were prepared in Mueller Hinton Broth (MHB) and incubated at 37 °C for 3–5 h. The turbidity of bacterial cell suspension was calibrated to 0.5 McFarland Standard by spectrophotometric method (OD 625 nm 0.08–0.10), as indicated in CLSI protocol M7-A9 [39] and, further, the standardized suspension was diluted (1:100) with MHB to reach 1–2 × 10^6^ CFU/mL. All wells were seeded with 20 µL of the mentioned final inoculum and some wells contained only inoculated broth as control growth. The plates were incubated at 37 °C for 24 h, and the MIC values were recorded as the lowest concentration of compounds at which there was no optically detectable microorganism growth and evaluated by comparing the growth in every well visually with that of the growth control well for bacteria. The MICs were determined by using the assay repeated twice in triplicate. For compound **2bG**, we observed that MIC values did not vary at 24, 48 and 72 h, suggesting bactericidal activity. Thus, from all the wells where no visible bacterial growth was detected, aliquots of 50 μL were taken, seeded on Mueller Hinton Agar (MHA) plates and incubated for 24 h at 37 °C. According to CLSI M26A guidelines [44], the absence of growth for bacterial strains confirmed the bactericidal activity of this compound. Throughout the study, norfloxacin was used as reference antibiotic. Appropriate controls involved norfloxacin for bacteria as international reference standard. In addition, DMSO alone (used as diluent of tested compounds) was tested for its activity against bacteria and consequently quantities of this solvent under 5% concentration were used during the execution of the experiments. Each species was tested at least three times in duplicate [40]. The microtiter broth microdilution (MBM) assay for bacteria was based on the recommended procedures by CLSI [39].

### 3.3. Cell Cultures

The MCF-10A human mammary epithelial cells were purchased from the American Type Culture Collection (ATCC, Manassas, VA, USA). They were cultured in Dulbecco’s modified Eagle’s medium/nutrient mixture Ham F-12 (DMEM/F12), supplemented with 5% horse serum (HS, Thermo Fisher Scientific, Milan, Italy), 100 U mL^−1^ penicillin/streptomycin, 0.5 mg mL^−1^ hydrocortisone, 20 ng mL^−1^ human epidermal growth factor (hEGF), 10 mg mL^−1^ insulin and 0.1 mg mL^−1^ cholera enterotoxin (Sigma-Aldrich, Milan, Italy). Cells were maintained at 37 °C in a humidified atmosphere of 95% air and 5% CO_2_ and periodically screened for contamination [45].

### 3.4. Cell Viability

Cell viability was determined using the 3-(4,5-dimethylthiazol-2-y1)-2,5-diphenyltetrazolium bromide (MTT, Sigma-Aldrich, Milan, Italy) assay [46]. Cells were seeded on 48-well plates and grown in complete medium. Before being treated, cells were starved in serum-free medium for 24 h to allow cell cycle synchronization. Then, cells were treated in phenol-red-free medium supplemented with 1% of serum with increasing concentrations of each compound for 24 h (4, 8, 16, 32 and 100 µg mL^−1^) and after fresh MTT was added to each well (final concentration 0.5 mg/mL). After 2 h incubation at 37 °C, cells were lysed with DMSO, and then optical density was measured at 570 nm using a microplate reader. For each sample, the mean absorbance was expressed as a percentage over the control and plotted versus drug concentrations to determine the IC_50_ values by using GraphPad Prism 9 software (GraphPad Inc., San Diego, CA, USA). Data are representative of three independent experiments; standard deviations (SD) have been reported.

## 4. Conclusions

The increase in bacterial infections that are resistant to almost all known antibiotics is alarming and becoming a major problem worldwide. TCC is an antibacterial agent widely used years ago and then retired from the market in 2016. In the search for new compounds endowed with antibacterial activity that may substitute TCC, a series of diarylureas was synthesized and the obtained results strengthened our previous observations that the substitution of the 3,4-dichloro aryl moiety of TCC with a 2,6 xylyl one enhances the antibacterial activity. Particularly, we showed that the introduction of a benzothiazole group increased the antibacterial activity in compounds bearing a 2,6 xylyl or a 3,4-dichlorophenyl group. We found that the most active compounds were **2eC**, which is the benzothiazole analogue of TCC (i.e., with the 3,4-dichlorophenyl and the 6-chloro-1,3-benzothiazole moieties), and **2bF** (MIC = 8 µg/mL *versus* 16 µg/mL of TCC), which bears the 2,6-xylyl and the 5-chloro-6-fluoro-1,3-benzothiazol-2-yl moieties. Interestingly, the 2,6-xylylderivative **2bB** was four-times more active than TCC against *E. faecalis*. The easy and cheap synthesis procedure adopted to obtain these small molecules and the improved antibacterial activity with respect to TCC, together with the absence of cytotoxicity on the normal cells, were the main goals of these studies. We are confident that the presented outcomes may pave the way for further studies of this interesting class of compounds.

## Figures and Tables

**Table 1:** Antibacterial activity of diarylureas.

		Microorganisms (MIC, µg/mL)						
		Gram-Positive					Gram-Negative	
Compd	Structure	*S.a.* 25923	*S.a.* 29213	*S.a.* 6538	*S.a.* 6538p	*E.f.* 29212	*P. a.* 27853	*K.p.* 13883
**2eA**	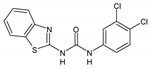	nt	64	32	16	32	>256	>256
**2eB**	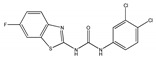	nt	64	128	A.N.	32	>256	A.N.
**2bB**	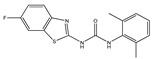	nt	16	128	A.N.	8	>256	A.N.
**2cC**	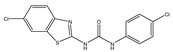	A.N.	>512	A.N.	>512	A.N.	A.N.	A.N.
**2bC**	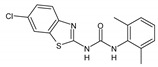	128	128	64	64	256	>512	>512
**2bF**	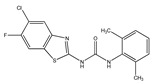	32	32	8	8	32	>512	>512
**2eE**	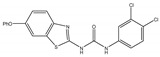	16	16	16	16	64	>512	>512
**2eC**	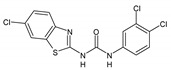	16	16	8	8	32	>512	>512
**2dC**	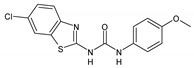	512	512	256	128	128	>512	>512
**2bG ^a^**	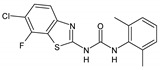	32	32	64	64	32	>512	>512
**NRF ^b^**		-	0.5–2	nt	nt	2–8	1–4	nt
**TCC**		16	16	128	16	64	256	256

Antibacterial activity was assessed using the CLSI assay (2012) [39]. Abbreviations: *S.a.*: *S. aureus*; *E.f.*: *E. faecalis*; *P.a.*: *P. aeruginosa*; *K.p.*: *K. pneumoniae*. NRF: norfloxacin; TCC: triclocarban; nt: not tested; ^a^ bactericidal action; ^b^ Values according to CLSI (2012) [39].

## Data Availability

The data presented in this study are available in manuscript.

## Abbreviations

ABAD: amyloid beta-binding alcohol dehydrogenase; AD, Alzheimer’s disease; BTA, benzothiazole; CEC, Contaminant of Emerging Concern; CLSI, Clinical and Laboratory Standards Institute; DMSO, dimethyl sulfoxide; hEGF, human epidermal growth factor; FBS, fetal bovine serum; HS, horse serum; IE, infective endocarditis; IR, infrared; LC–MS, liquid chromatography–mass spectrometry; MBM, microtiter broth microdilution; MEM, minimum essential medium; MHA, Mueller Hinton Agar; MICs, minimum inhibitory concentrations; MTT, 3-(4,5-Dimethylthiazol-2-y1)-2,5-diphenyltetrazolium bromide; NMR, nuclear magnetic resonance; NRF, norfloxacin; PD, Parkinson’s disease; SD, standard deviations; TCC, triclocarban.
